# Hemodialysis-related allergic reactions: a retrospective cohort study of clinical presentations, etiologies, and management

**DOI:** 10.1186/s12882-026-05075-w

**Published:** 2026-06-03

**Authors:** Jing-yao Lu, Qi Ke, Jun Li, Wen-qing Lv, Ruo-yu Wang, Chun-qing Li

**Affiliations:** 1 Department of Nephrology, Affiliated Hospital of Jiangnan University, No. 1000, He Feng Rd., Wuxi, Jiangsu 214122 China; 2 Wuxi School of Medicine, Jiangnan University, No. 1800, Li hu Rd., Wuxi, Jiangsu 214122 China

**Keywords:** Hemodialysis, End-stage renal disease, Allergic reactions, Ethylene oxide, Dialyzer hypersensitivity reaction, Retrospective analysis

## Abstract

**Objective:**

This study aimed to investigate the etiology, clinical manifestations, management strategies, and clinical outcome of hemodialysis-related allergic reactions.

**Methods:**

A retrospective cohort study was conducted to analyze clinical data from patients who experienced hemodialysis-related allergic reactions at the Affiliated Hospital of Jiangnan University between January 2020 and December 2025. Patients undergoing maintenance hemodialysis—with no prior history of these allergic reactions—served as the control group. Their clinical characteristics and reaction profiles were systematically analyzed.

**Results:**

A total of 40 cases of hemodialysis-related allergic reactions were identified as the study group. Isolated pruritus and skin rash were observed in 25 patients (62.5%); hemodialysis-related leukopenia and thrombocytopenia, in 4 cases (10.0%); and severe anaphylactic reactions, in five cases (12.5%); Five cases exhibited atypical hemodialysis-related allergic reactions (12.5%), including hypotension within 10 min of hemodialysis initiation, malaise, nausea, and vomiting. The predominant etiological factors identified were hypersensitivity to the hemodialyzer membrane (*n* = 11, 27.5%), AVF needles kits and hemodialysis extracorporeal circuit tubing (Ethylene Oxide Residue) (*n* = 27, 67.5%), and Nafamostat Mesylate (*n* = 2, 5%). Thorough saline pre-rinsing of the extracorporeal circuit remained the primary preventive strategy against EO-induced hypersensitivity. Switching to irradiated-sterilized acetate-membrane or wet-membrane dialyzers effectively prevented dialyzer hypersensitivity reaction. Glucocorticoid administration was effective in mitigating severe anaphylactic reactions. Patients in the study group exhibited significantly higher plasma eosinophil counts and C-reactive protein concentrations, and significantly lower hemoglobin levels, and serum albumin concentrations compared with the control group.

**Conclusion:**

Hemodialysis-related allergic reactions are primarily attributable to hypersensitivity responses to dialyzer membranes, residual ethylene oxide in extracorporeal circuit components, and occasionally Nafamostat Mesylate administered during the procedure. Glucocorticoid administration was effective in attenuating severe anaphylactic reactions. Allergic reactions that repeatedly occur during hemodialysis may be associated with an underlying chronic micro-inflammatory state.

## Introduction

According to data from the Chinese Research Data Services Platform (CNRDS), it is estimated that approximately 5.4 million individuals globally with end-stage renal disease (ESRD) will receive kidney replacement therapy by 2030 [1]. Hemodialysis remains the predominant form of life-sustaining renal replacement therapy for individuals with ESRD, representing 82.4% of incident cases reported in 2022 [2]. Hemodialysis effectively removes accumulated metabolic waste products and uremic toxins and helps maintain fluid, electrolyte, and acid-base homeostasis by leveraging the physicochemical principles of diffusion, convection, and adsorption across a semipermeable dialyzer membrane. However, allergic reactions occurring during hemodialysis not only compromise treatment efficacy but may also pose life-threatening risks in severe cases [3].

The immunological mechanisms underlying dialyzer hypersensitivity reaction (DHR) are broadly classified into two categories: IgE-mediated type I hypersensitivity (designated Type A) and non-IgE-mediated allergic reactions (designated Type B) [4]. Type A DHR are triggered by specific allergenic stimuli—including ethylene oxide (EO)-protein adducts, leachables, and degradation products released from dialyzer components—which promote Th2 lymphocyte differentiation and subsequent interleukin-4 (IL-4) secretion [5]. IL-4 drives B-cell class-switching to produce allergen-specific immunoglobulin E (IgE). This IgE binds with high affinity to FcεRI receptors expressed on mast cells and basophils, resulting in cellular degranulation and the release of inflammatory mediator—including histamine, tryptase, leukotrienes, and prostaglandins. Tryptase further activates the complement and kallikrein–kinin systems, thereby amplifying the inflammatory cascade [6]. Concurrently, Th2 cells and activated mast cells secrete interleukin-5 (IL-5), a key cytokine that promotes eosinophil recruitment, differentiation, activation, and degranulation—resulting in the release of cytotoxic granule proteins, including eosinophil cationic protein, eosinophil peroxidase, and eosinophil-derived neurotoxin [7]. Collectively, these inflammatory mediators induce characteristic clinical manifestations: bronchoconstriction (causing dyspnea), increased vascular permeability (leading to angioedema and hypotension), and sensory nerve stimulation (resulting in pruritus).

In contrast, type B DHR are primarily triggered by contact activation of the complement system mediated by the dialyzer membrane surface. Complement cleavage generates anaphylatoxins C3a and C5a, which directly stimulate mast cells and basophils—eliciting mediator release and downstream effects phenotypically similar to those observed in Type A reactions [8, 9]. Moreover, certain negatively charged dialyzer membranes—particularly polyacrylonitrile (AN69)—can activate the intrinsic coagulation pathway, especially in patients receiving angiotensin-converting enzyme inhibitors (ACEIs). This activation promotes bradykinin generation and accumulation, a potent endogenous vasodilator that markedly increases vascular permeability and thereby precipitates hypotension and life-threatening angioedema [10].

Furthermore, recurrent and prolonged weekly exposure of blood to the hemodialyzer and extracorporeal circuit—typically three times per week—may activate the complement system and induce a state of chronic microinflammation [8], thereby triggering intravascular inflammatory cascades and accelerating atherosclerotic progression, which collectively elevate the risk of cardiovascular events in patients undergoing maintenance hemodialysis [11]. This study reports a retrospective review of 40 documented cases of hemodialysis-associated hypersensitivity reactions identified at the Affiliated Hospital of Jiangnan University between January 2020 and December 2025. A comprehensive literature review was conducted to synthesize current evidence regarding the potential allergens, clinical manifestations, therapeutic approaches, and clinical outcomes associated with allergic reactions during hemodialysis, thereby supporting evidence-informed diagnosis and management in clinical practice.

## Materials and methods

### The patients enrolled

A retrospective cohort study was conducted involving 40 adult patients undergoing hemodialysis who experienced hemodialysis-related allergic reactions at the Affiliated Hospital of Jiangnan University between January 2020 and December 2025(the study group). A control group comprising 40 patients undergoing maintenance hemodialysis during the same period was established; all individuals were matched to the case group by age and sex and had no documented history of hemodialysis-associated hypersensitivity reactions. The diagnosis of chronic kidney disease stage 5 was confirmed according to KDIGO 2021 Clinical Practice Guideline. Patients were excluded if they met any of the following criteria: concurrent receipt of hemodialysis and peritoneal dialysis; hematologic malignancy; sepsis; operation within one month; pre-existing eosinophilia, pre-existing leukopenia, pre-existing thrombocytopenia (documented prior to initiation of hemodialysis); acute asthma exacerbation; or non–hemodialysis-related allergic dermatoses—including atopic eczema, insect bite–induced dermatitis, or chronic urticaria. The study protocol was approved by the Medical Ethical Review Committee of the Affiliated Hospital of Jiangnan University (LS2025417).

#### The definition of hemodialysis-related allergic reactions

the allergic reactions may recur upon re-exposure to the same dialyzer, extracorporeal circuit tubing, or vascular access needle in subsequent hemodialysis sessions. Clinical manifestations range from mild symptoms—including urticaria, pruritus, abdominal cramps, nausea, and vomiting—to severe systemic reactions such as chest tightness, dyspnea, hypoxemia (as indicated by decreased oxygen saturation), and hypotension [4, 12–13, 14–15].

#### The definition of dialyzer hypersensitivity reaction (DHR)

DHR are classified into Type A (immediate-onset) and Type B (delayed-onset) reactions. Type A reactions occur within 5–30 min of dialysis initiation, with rapid onset of severe, systemic symptoms including chest discomfort, back pain, diaphoresis, dyspnea, hypoxemia (evidenced by decreased oxygen saturation), and hypotension. Type B hypersensitivity reactions typically emerge 20 to 40 min—or later—following the initiation of hemodialysis. Clinical presentation includes urticaria, chest pain, back pain, and mild dyspnea. Diagnosis of hemodialyzer-associated hypersensitivity necessitates the systematic exclusion of alternative etiologies, such as dialysis-induced hypotension, dialysis disequilibrium syndrome, acute coronary syndrome, and cerebrovascular accidents [4, 12].

### Severity grading scale for anaphylaxis [14–15]

Grade I: Characterized by generalized cutaneous and mucosal symptoms. This includes erythema (redness), flushing, pruritus (itching), and urticaria (hives), and may also include angioedema. Grade II: Involves moderate, multi-organ symptoms that are measurable but not immediately life-threatening. This grade adds symptoms from other systems on top of the skin signs. It can include gastrointestinal disturbances (nausea, cramping), respiratory symptoms (rhinorrhea, dyspnea), and cardiovascular signs (tachycardia > 20 bpm change, hypotension > 20 mmHg change but not profound). Grade III: Defined by severe, life-threatening, multi-organ involvement. This grade signifies a critical state and requires immediate, aggressive intervention. It is characterized by severe respiratory distress (bronchospasm, cyanosis) or circulatory failure, including shock, severe hypotension, and arrhythmias. Consciousness is often impaired. Grade IV: The most severe grade, representing cardiac and/or respiratory arrest. This is a state of vital function cessation requiring full cardiopulmonary resuscitation.

### Statistical analysis

Statistical analysis was performed using SPSS software (version 26.0; IBM Corporation, Armonk, NY, USA). Propensity score matching was conducted on a screening cohort of 280 patients to create balanced comparison groups. Key covariates incorporated into the matching model were age, sex, dialysis vintage, Kt/V, serum calcium and phosphorus levels, parathyroid hormone (PTH) concentration, ferritin level, medical history of diabetes, hypertension, and cardiovascular disease. Using a 1:1 nearest-neighbor algorithm without replacement, we generated a final matched sample comprising 40 patients in the study group and 40 in the control group. Continuous variables with normal distribution were expressed as mean ± standard deviation and compared using independent samples t-test, while non-normally distributed variables were presented as median (interquartile range) and compared using Mann-Whitney U test. Categorical variables were expressed as number (percentage) and compared using chi-square test or Fisher’s exact test, as appropriate. All statistical tests were two-sided, and a p-value < 0.05 was considered statistically significant. Effect sizes (such as mean differences or odds ratios) with corresponding 95% confidence intervals were calculated for all primary comparisons to provide clinical context beyond p-values. Missing data were minimal (< 5%) and handled using complete case analysis. All statistical analyses followed current reporting guidelines for observational studies (STROBE statement).

## Results

1. Clinical characteristics of the enrolled patients.

A total of 78,474 hemodialysis sessions were performed during the study period; of these, 36,451 occurred during the peak period (January 2024-December 2025). The estimated incidence of hemodialysis-related allergic events ranged from 5 to 11 per 10,000 dialysis sessions. Study participants underwent hemodialysis using dialyzers equipped with either gamma-irradiated polysulfone or polyethersulfone membranes. All participants maintained vascular access via an autogenous arteriovenous fistula, except for two individuals who required internal jugular vein catheterization. No participant was administered an angiotensin-converting enzyme inhibitor (ACEI). The comparison of clinical characteristics between the patients with hemodialysis-related allergic reaction (the study group) and those without the allergic reaction (the control group) are summarized in Table 1. No significant differences were observed in the proportions of patients receiving ARBs or beta-blockers between the two groups. No statistically significant differences were observed between the two groups with respect to the prevalence of hypertension, diabetes, or prior cardiovascular and cerebrovascular disease.

**Table 1:** The comparison of clinical characteristics between the patients in the study group and the control group

Character	The study group (*n* = 40)	The control group (*n* = 40)	*P* Value	95% Confidence interval of the Difference	
				Lower	Upper
Age(y)	61 ± 13	57 ± 15	0.228	-2.4543	10.1543
Dialysis vintage (y)	2.7(1.8,7.7)	2(1.2, 3.6)	0.142	-0.54132	3.70282
Eosinophils(10^9^/µl)	0.9(0.4,1.5)	0.2(0.1, 0.3)	0.017	0.23689	2.22311
Eosinophil Percentage (%)	11.4(8.4,19.9)	2.2(1.7, 3.7)	0.0001	8.53359	16.07641
Basophil Percentage (%)	0.8 ± 0.4	0.4 ± 0.2	0.335	0.19181	0.47819
CRP (mg/l)	5.0(3.2, 10.4)	3.1(2.7,3.5)	0.0001	2.26156	6.61344
Calcium(mmol/l)	2.2 ± 0.2	2.2 ± 0.1	0.399	-0.10895	0.4395
Phosphorus(mmol/l)	1.7 ± 0.4	1.7 ± 0.5	0.423	-0.26372	0.11172
iPTH (pg/ml)	183(91,269)	165(97,275)	0.226	-23.15218	96.64218
Hemoglobulin(g/l)	101 ± 21	119 ± 12	0.0001	-25.81887	-10.73113
Ferritin(ng/ml)	77(44,143)	78(40,165)	0.245	-28.84494	111.37994
Platelet(10^9^/µl)	169 ± 72	191 ± 44	0.110	-48.29152	5.04152
CHOL(mmol/l)	3.6 ± 0.9	3.7 ± 1.0	0.725	-0.51842	0.36242
LDL-C(mmol/l)	1.8 ± 0.7	1.7 ± 0.7	0.554	-0.21441	0.39691
HDL-C(mmol/l)	0.9 ± 0.3	0.9 ± 0.2	0.400	-0.06696	0.16596
TG (mmol/l)	1.0(0.8,2.1)	1.5(1.0, 2.8)	0.042	-0.92748	-0.01752
Albumin(g/l)	39.5 ± 3.8	41.5 ± 2.6	0.005	-3.57842	-0.64658

Note: CRP C-reactive protein, iPTH intact parathyroid hormone; CHOL Cholesterol; LDL-C low-density lipoprotein cholesterol; HDL-C high-density lipoprotein cholesterol; TG triglycerides

**Fig. 1:**
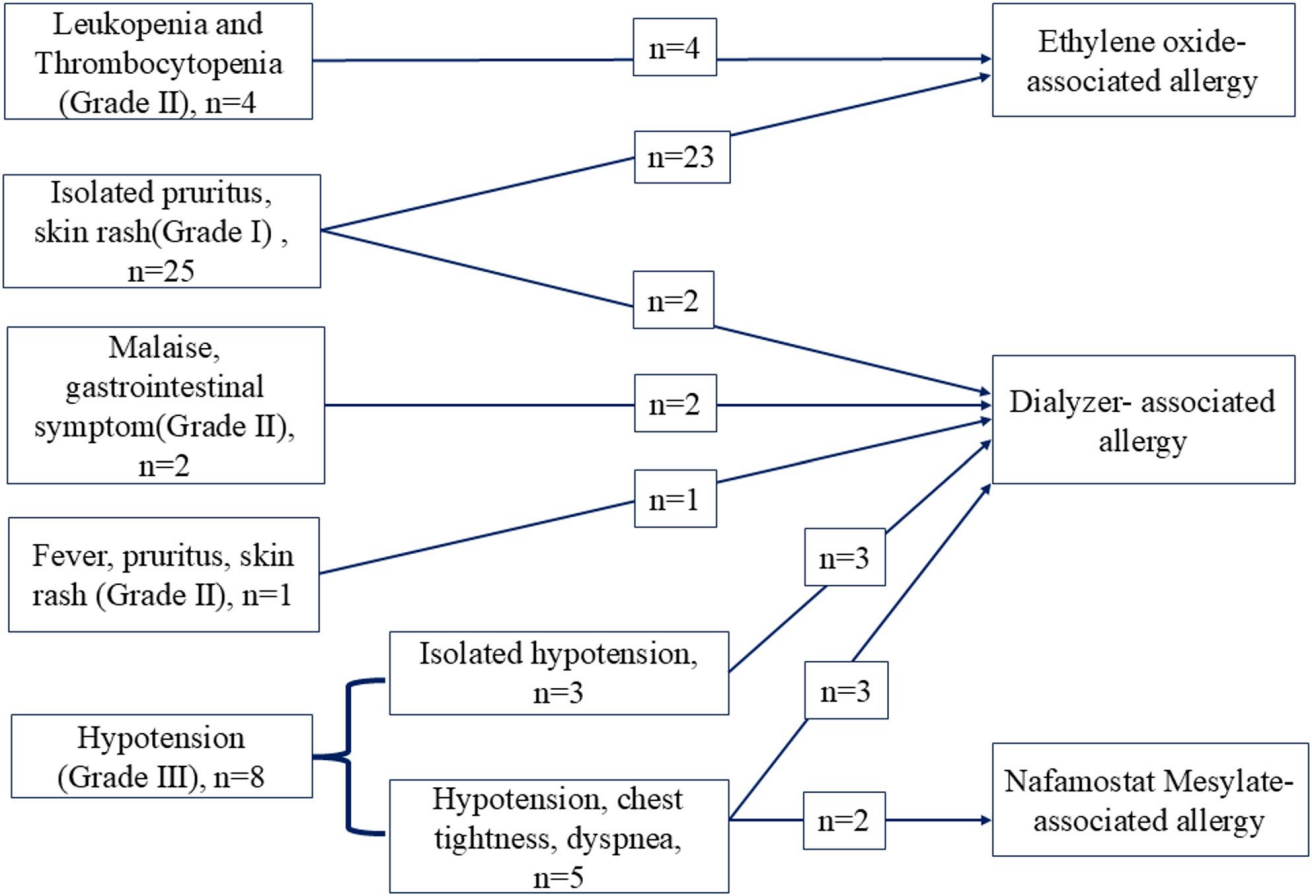
Schematic summary of clinical manifestations and putative allergenic triggers in 40 patients with hemodialysis-associated allergic reactions

2. Clinical manifestations, management strategies, and outcomes among individuals experiencing hemodialysis-associated hypersensitivity reactions (Tables 2 and 3; Fig. 1).

**Table 2:** Clinical manifestation of hemodialysis-associated allergic reactions

Clinical manifestation	Number	Percentage(%)
Chest tightness and dyspnea	5	12.5
Skin rash and pruritus	30	75
Malaise and gastrointestinal symptom	2	5
Hypotension	8	20
Leukopenia and Thrombocytopenia	4	10
Fever	1	2.5

**Table 3:** Putative allergenic triggers of hemodialysis-associated allergic reactions

Putative Allergenic Triggers	Number	Percentage(%)
Dialyzer with polysulfone membranes	10	25
Dialyzer with polyethersulfone membrane	1	2.5
AVF needles kits and hemodialysis circuits tubing (Ethylene Oxide Residue)	27	67.5
Nafamostat Mesylate	2	5

Note: Autologous arteriovenous fistula

Patients in the study group exhibited significantly higher plasma eosinophil counts and C-reactive protein concentrations, and significantly lower hemoglobin levels, serum albumin concentrations, and triglyceride levels compared with the control group.

Among the 40 cases of the study group, 25 cases presented with isolated pruritus accompanied by localized cutaneous rashes—predominantly affecting the upper limb containing the arteriovenous fistula (AVF)—with symptom exacerbation during hemodialysis and resolution during the interdialytic interval. In two cases, the rash was more severe and generalized and improved following replacement of the hemodialyzer with either a cellulose triacetate membrane or a wet-membrane dialyzer. In the other 23 cases, with the same dialyzer type retained, replacement of the AVF needle combined with thorough saline pre-rinsing of the extracorporeal circuit tubing led to marked symptom alleviation.

Five patients experienced severe anaphylactic reactions. Two patients exhibited a classic type A DHR. The remaining three patients, all undergoing maintenance hemodialysis for more than one year, developed the same systemic symptoms accompanied by palmoplantar pruritus approximately 30 min after hemodialysis commencement. Hemodialysis was immediately discontinued in all cases, and the blood contained within the extracorporeal circuit was discarded. Intravenous dexamethasone administration led to gradual symptom resolution. Among these five cases, one patient—undergoing hemodialysis for the first time—experienced recurrent severe anaphylactic reactions despite switching to a cellulose acetate membrane dialyzer; hemodialysis was therefore discontinued, and the patient was transitioned to peritoneal dialysis. In two cases, cessation of Nafamostat Mesylate resolved the symptoms, permitting successful resumption of hemodialysis. In the final two cases, no further allergic reactions occurred following replacement of the hemodialyzer. And two of these five also demonstrated increased plasma IgE concentrations, who were highly suggestive of type A dialyzer hypersensitivity reaction. Serum complement C3 and C4 concentrations were measured in all five cases and fell within the reference (normal) range.

Another five cases exhibited atypical hemodialysis-related allergic reactions in the absence of ultrafiltration. In three patients, hypotension developed within 10 min of hemodialysis initiation in the absence of ultrafiltration; potential non-allergic causes of intradialytic hypotension—including acute myocardial infarction, hypovolemia, and hypoglycemia—were rigorously excluded. The remaining two patients developed malaise, nausea, and vomiting approximately one hour after treatment commencement; and dialysis disequilibrium syndrome were excluded. Following transition to a hemodialyzer incorporating either a cellulose triacetate membrane or a wet-membrane, all procedures proceeded without further adverse events.

Four patients developed hemodialysis-related leukopenia and thrombocytopenia; two of these additionally exhibited cutaneous rash and pruritus. Bone marrow aspiration demonstrated no evidence of primary hematologic malignancy or dysplasia, infectious indicators was negative, and no antiplatelet agents had been administered. Laboratory evaluation of anemia-related parameters confirmed that serum folate and vitamin B_12_ concentrations were within normal reference ranges.

## Discussion

Hemodialysis-related allergic reactions are a well-recognized complication of maintenance hemodialysis. The pathophysiological basis of these hypersensitivity reactions involves immune activation triggered by blood exposure to extracorporeal circuit components—particularly synthetic or modified cellulose dialysis membranes and associated tubing [3, 16]. Established etiologies include immunoglobulin E (IgE)-mediated or complement-dependent hypersensitivity to dialyzer membrane materials [9]; residual ethylene oxide—a sterilant used in the manufacturing of AVF needles, cannulas, and hemodialysis circuits [13]; and pharmacologic agents administered during the hemodialysis session, such as heparin, Nafamostat Mesylate, or iron preparations [17–18].

Our study reports five cases of severe anaphylactic reactions associated with hemodialysis: three highly suggestive of hemodialyzer-induced hypersensitivity, and two were attributed to Nafamostat Mesylate hypersensitivity. All patients exhibited peripheral eosinophilia; serum IgE levels were elevated in two cases of DHR. Serum complement C3 and C4 levels were within reference ranges across all five cases. Two cases manifested classic type A FUS, with symptom onset within five minutes of hemodialysis initiation. Although the remaining three cases presented approximately 30 min after treatment commencement, their severe, systemic anaphylactic phenotype—characterized by rapid hemodynamic compromise and respiratory distress—led to clinical classification as type A FUS for urgent management purposes. These findings support the development of a refined, mechanism-based classification system for hemodialysis-related anaphylaxis to inform targeted interventions and avoid serious adverse events.

Additionally, three patients developed atypical hemodialyzer-associated allergic reactions, characterized by intradialytic hypotension occurring within 10 min of hemodialysis initiation in the absence of ultrafiltration. In one patient—following two prior hemodialysis sessions using an identical polysulfone membrane dialyzer sterilized by gamma irradiation—hypotension and hypoxemia (evidenced by decreased oxygen saturation) developed within minutes of treatment commencement. Notably, in previous dialysis sessions, identical hypotensive episodes had been managed successfully with intravenous saline infusion and 50% dextrose solution administration, enabling blood pressure stabilization and completion of the procedure. This pattern increases the risk of misclassifying such reactions as non-allergic, volume-mediated hypotension—potentially leading to underdiagnosis of hemodialysis-related allergy. These findings align with those reported by Fukumi et al. [19]. A diagnostic trial involving hemodialyzer replacement—followed by symptom resolution—may support clinical suspicion of hemodialyzer-related hypersensitivity. Crucially, alternative causes of intradialytic hypotension—including hypovolemia, dialysis disequilibrium syndrome, excessive ultrafiltration, and acute cardiovascular events—must be systematically excluded [20, 21]. In our cohort, serial monitoring of cardiac injury biomarkers and electrocardiographic parameters failed to fulfill the diagnostic criteria for acute coronary syndrome. Echocardiography excluded structural and functional cardiac abnormalities, including valvular heart disease and pulmonary hypertension.

Severe hemodialysis-related anaphylactic reactions are most frequently attributable to hypersensitivity to dialyzer membrane materials. Dialyzer membranes are broadly categorized into cellulose-based membranes (e.g., cuprophane) and synthetic membranes (e.g., polysulfone, polyethersulfone). Both classes can activate the complement system—predominantly via the alternative and lectin pathways—leading to generation of the anaphylatoxins C3a and C5a. These mediators induce vascular smooth muscle contraction, increase microvascular permeability, and stimulate histamine release from mast cells and basophils [22, 23]. Clinical evidence further supports a distinct immunopathological profile in polysulfone hypersensitivity: comparative studies demonstrate that patients with confirmed polysulfone allergy—relative to those with cellulose triacetate membrane sensitivity—exhibit significantly greater basophil degranulation, T-cell activation, elevated serum tryptase concentrations, and pronounced depletion of complement components C3 and C4 [5, 22, 24]. Consistent with it, multiple observational studies have consistently demonstrated that a subset of patients undergoing maintenance hemodialysis exhibit reduced circulating complement component levels [8, 25]. In our study, DHR were predominantly associated with polysulfone membranes; only one case involved polyethersulfone membrane hypersensitivity.

Cellulose triacetate membranes are synthesized via acetylation of native cellulose, conferring favorable biocompatibility and enhanced hydrophilicity [5, 26]. Wet-membrane dialyzers—pre-hydrated with sterile water or a manufacturer-specified solution prior to clinical use—represent a distinct design strategy aimed at optimizing hemocompatibility [23]. In the present cohort, hemodialyzer-associated anaphylactic reactions resolved in all but one case (transit to peritoneal dialysis) following transition to either cellulose triacetate membrane or wet-membrane dialyzers.

Ethylene oxide (EO) residue remains a well-documented cause of hemodialysis-related hypersensitivity reactions [13]. EO is a highly reactive alkylating agent widely employed for the terminal sterilization of heat-labile medical devices. Laboratory investigations in affected patients consistently demonstrate marked leukocytosis, peripheral eosinophilia, and elevated serum levels of EO-specific IgE antibodies. As a low-molecular-weight hapten, EO covalently binds to endogenous carrier proteins—such as albumin or immunoglobulins—to form neoantigens, initiating T lymphocyte activation and subsequent B-cell differentiation into EO-specific IgE-secreting plasma cells. The resulting IgE antibodies bind with high affinity to FcεRI receptors on mast cells and basophils. Upon re-exposure to EO-derived neoantigens, cross-linking of membrane-bound IgE triggers effector cell degranulation, releasing preformed mediators—including histamine—and newly synthesized inflammatory effectors such as proteolytic enzymes and cytokines, thereby propagating the allergic cascade [27]. Although gamma irradiation has largely replaced EO for dialyzer sterilization—thereby markedly reducing EO-related reactions—EO remains the predominant sterilant for ancillary extracorporeal components, including dialysis tubing, needles, and vascular cannulae. Consequently, rigorous saline pre-rinsing of the entire extracorporeal circuit prior to patient connection is the cornerstone preventive strategy to eliminate residual EO and mitigate the risk of intra-dialytic hypersensitivity. Fortunately, Omalizumab—a humanized monoclonal anti-IgE antibody—exerts its promising therapeutic effect in DHR primarily through dual mechanisms: (1) high-affinity neutralization of free IgE, thereby preventing IgE binding to FcεRI on effector cells; and (2) downregulation of FcεRI expression on mast cells and basophils [28]. Supporting this, case reports document rapid and sustained resolution of ethylene oxide–induced hypersensitivity following omalizumab administration in hemodialysis patients [13].

A total of 27 patients in this cohort exhibited clinical features consistent with hypersensitivity reactions strongly associated with EO residue contamination in arteriovenous fistula (AVF) needles or hemodialysis circuit tubing; all cases were accompanied by peripheral eosinophilia [13]. Moreover, polyvinyl chloride or other polymer-based plastics containing leachable plasticizers, including di(2-ethylhexyl) phthalate may induce type IV delayed hypersensitivity and, in susceptible individuals, to promote IgE-mediated allergic responses [29]. However, no standardized diagnostic method is currently available for this type of allergy.

Nafamostat Mesylate is particularly suitable for regional anticoagulation in extracorporeal circuits, especially in patients at elevated risk of bleeding or with active hemorrhage. However, anaphylactic reactions to Nafamostat Mesylate have been documented in rare but clinically significant cases [18]. In patients with confirmed Nafamostat Mesylate hypersensitivity, skin testing—including the skin prick test and intradermal test—as well as the basophil activation test, consistently yields positive results during diagnostic evaluation. In contrast, serum-specific IgE testing exhibits low sensitivity and insufficient diagnostic accuracy for Nafamostat Mesylate-induced hypersensitivity [30]. Among the two cases of Nafamostat Mesylate hypersensitivity described herein, one patient had undergone two prior renal transplantations and subsequently discontinued immunosuppressive therapy; the other had systemic lupus erythematosus. Both patients presented with peripheral eosinophilia, yet serum total IgE concentrations, as well as complement C3 and C4 levels, remained within normal reference ranges. These findings suggest that measurement of Nafamostat Mesylate-specific IgE antibodies—rather than total IgE—may offer greater diagnostic utility in suspected Nafamostat Mesylate hypersensitivity.

In this cohort, four cases presented with concurrent thrombocytopenia and leukopenia. These hematologic abnormalities were definitively excluded as heparin-induced thrombocytopenia, based on negative anti-PF4/heparin antibody testing and absence of clinical HIT criteria [17]. A more plausible mechanism involves complement activation secondary to repeated and prolonged blood–membrane interaction—whether mediated by dialyzer membrane materials or residual EO (which drives platelet activation, aggregation, adhesion, and consumption [9]. Complement-derived anaphylatoxins—particularly C5a and C3a—further promote neutrophil and monocyte activation, induce NETosis with subsequent neutrophil extracellular trap (NET) formation [31], and stimulate the release of reactive oxygen species, proteolytic enzymes, and pro-inflammatory cytokines—collectively contributing to sustained peripheral leukocyte depletion [8, 17]. Moreover, direct leukocyte adhesion to the hemodialysis membrane surface during extracorporeal circulation may facilitate pulmonary leukostasis, a recognized complication associated with acute respiratory distress in susceptible patients [32].

Post-hemodialysis fever warrants inclusion of hemodialysis-related hypersensitivity reactions in the differential diagnosis. Eosinophilia and elevated levels of IgE represent the potential biomarkers for identifying hemodialysis-related allergic reaction [33]. In the present case, the alternative etiologies for fever—including infection, autoimmune disease, and malignancy—as rigorously excluded by comprehensive clinical and laboratory evaluation. Furthermore, the febrile episode resolved promptly following replacement of the hemodialyzer, supporting a causal association with the extracorporeal circuit component.

Our study demonstrates that maintenance hemodialysis patients with hemodialysis-related allergic reactions demonstrated significantly elevated peripheral eosinophil counts and C-reactive protein concentrations. Recurrent hemodialysis-associated hypersensitivity reactions may promote a chronic low-grade inflammatory state. Whether this inflammation contributes to elevated cardiovascular events in the affected population remains to be elucidated. Although serum tryptase measurement, cutaneous testing, the basophil activation test, and assays for EO-specific IgE antibodies have been reported in the literature, none of these modalities is currently incorporated into routine diagnostic workflows for hemodialysis-related hypersensitivity reactions.

Collectively, obligatory contact between circulating blood and synthetic surfaces—including dialyzer membranes, tubing, needles, and connectors during hemodialysis—triggers hemodialysis-related allergic reactions. They are primarily attributable to hypersensitivity responses to dialyzer membranes, residual ethylene oxide in extracorporeal circuit components, and occasionally Nafamostat Mesylate administered during the procedure. Glucocorticoid administration was effective in attenuating severe anaphylactic reactions. Given its established efficacy in IgE-dependent hypersensitivity and favorable pharmacokinetic profile in renal impairment—owing to predominant reticuloendothelial system clearance rather than renal excretion—Omalizumab represents a mechanistically rational and clinically promising therapeutic option for dialyzer-related allergic reactions. Allergic reactions that repeatedly occur during hemodialysis may be associated with an underlying chronic micro-inflammatory state. There is a critical, unmet need for validated, disease-specific biomarkers to diagnose dialysis-associated allergic reactions. Furthermore, consensus guidelines are urgently required to standardize the definition, classification, and evidence-based management of these reactions—particularly to reduce the risk of life-threatening anaphylaxis and ameliorate the underlying microinflammatory burden.

## Limitation

This study is a single-center investigation with a limited sample size, thereby carrying inherent risks of selection and sampling bias. It also lacks validated biomarkers for the diagnosis of allergic reactions. Future research must prioritize the precise definition of dialysis-related allergic reactions, clinical validation of disease-specific diagnostic markers, and the development of stratified diagnostic and therapeutic strategies.

## Data Availability

The relevant data are available upon reasonable request from the corresponding author.

## Abbreviations

ESRD: End-stage renal disease
DHR: Dialyzer hypersensitivity reaction
ACEIs: Angiotensin-converting-enzyme inhibitor
IgE: Immunoglobulin E
FUS: First-use syndrome
EO: Ethylene oxide
AN69: Polyacrylonitrile
AVF: Autologous arteriovenous fistula
NET: Neutrophil extracellular trap
CRP: C-reactive protein
iPTH: Intact parathyroid hormone
CHOL: Cholesterol
LDL-C: Low-density lipoprotein cholesterol
HDL-C: High-density lipoprotein cholesterol
TG: Triglycerides
